# Chronic Exposure to Arsenic and Fluoride Starting at Gestation Alters Liver Mitochondrial Protein Expression and Induces Early Onset of Liver Fibrosis in Male Mouse Offspring

**DOI:** 10.1007/s12011-024-04198-1

**Published:** 2024-04-27

**Authors:** Wendy L. González-Alfonso, Pavel Petrosyan, Luz M. Del Razo, Luz C. Sánchez-Peña, Miguel Tapia-Rodríguez, Rolando Hernández-Muñoz, María E. Gonsebatt

**Affiliations:** 1 Departamento de Medicina Genómica y Toxicología Ambiental, Instituto de Investigaciones Biomédicas, Universidad Nacional Autónoma de México, Mexico City, 04510 México; 2 Departamento de Toxicología, Centro de Investigación y Estudios Avanzados, 07360 Mexico City, Mexico; 3 Unidad de Microscopia, Instituto de Investigaciones Biomédicas, Universidad Nacional Autónoma de México, 04510 Mexico City, Mexico; 4 Departamento de Biología Celular y del Desarrollo, Instituto de Fisiología Celular, Universidad Nacional Autónoma de México, Mexico City, 04510 México

**Keywords:** Arsenic, Fluoride, Liver damage, Mitochondria, Transsulfuration, Fibrosis

## Abstract

The presence of arsenic (As) and fluoride (F^−^) in drinking water is of concern due to the enormous number of individuals exposed to this condition worldwide. Studies in cultured cells and animal models have shown that As- or F-induced hepatotoxicity is primarily associated with redox disturbance and altered mitochondrial homeostasis. To explore the hepatotoxic effects of chronic combined exposure to As and F^−^ in drinking water, pregnant CD-1 mice were exposed to 2 mg/L As (sodium arsenite) and/or 25 mg/L F^−^ (sodium fluoride). The male offspring continued the exposure treatment up to 30 (P30) or 90 (P90) postnatal days. GSH levels, cysteine synthesis enzyme activities, and cysteine transporter levels were investigated in liver homogenates, as well as the expression of biomarkers of ferroptosis and mitochondrial biogenesis-related proteins. Serum transaminase levels and Hematoxylin–Eosin and Masson trichrome-stained liver tissue slices were examined. Combined exposure at P30 significantly reduced GSH levels and the mitochondrial transcription factor A (TFAM) expression while increasing lipid peroxidation, free Fe ^2+^, p53 expression, and serum ALT activity. At P90, the upregulation of cysteine uptake and synthesis was associated with a recovery of GSH levels. Nevertheless, the downregulation of TFAM continued and was now associated with a downstream inhibition of the expression of MT-CO2 and reduced levels of mtDNA and fibrotic liver damage. Our experimental approach using human-relevant doses gives evidence of the increased risk for early liver damage associated with elevated levels of As and F^−^ in the diet during intrauterine and postnatal period.

**Supplementary Information:**

The online version contains supplementary material available at 10.1007/s12011-024-04198-1.

## Introduction

Epidemiological and experimental studies have characterized the toxic effects of chronic exposure to arsenic (As) or fluoride (F^−^). Thus, groundwater pollution with inorganic As and F^−^ is still of concern due to the large number of individuals worldwide who drink water with high levels of these two elements [1]. For example, As and F^−^ levels in underground wells in Mexico can reach up to 0.87 mg/L and 23.4 mg/L, respectively [2], well over WHO (World Health Organization) recommended levels [3].

Liver damage, including hepatomegaly and hepatic cancer, has been associated with lifetime human exposure to As or F^−^ [4–6]. Since the liver is the primary organ responsible for xenobiotic detoxification [7], simultaneous exposure to both elements might lead to biochemical interactions affecting the toxic outcome.

In humans, as in most mammals, inorganic As (iAs) is methylated in the cell cytoplasm by the As 3 methyl transferase (AS3MT) enzyme in a process that requires S-adenosyl methionine (SAM) as a methyl donor and glutathione (GSH) as a reduction agent. As a result, in the liver, the As species distributed are iAs, monomethylated (MMA), dimethylated (DMA), and even trimethylated species of As as the trimethyl arsine oxide (TMAO) [8].

GSH (γ-L-glutamyl-L-cysteinylglycine) plays essential roles not only in the elimination of xenobiotics but also as the primary cell antioxidant. Its synthesis occurs in the cytosol and is distributed into different cell compartments [9]. GSH synthesis is limited by the availability of amino acids, mainly by L-cysteine, imported into cells by transport systems such as X^−^_AG_, ASC, Xc- and others. In hepatocytes, the solute carrier family1 member 4 (SLC1A4, also known as alanine-serine-cysteine transporter 1 (ASCT1)) is the predominant cysteine transporter [10]. Another cysteine source for GSH synthesis is supplied by converting L-methionine to L-cysteine in the transsulfuration pathway.

GSH pools show different kinetic responses under oxidative damage; for example, cytosolic pools recover faster than mitochondrial pools [11, 12]. GSH import into the mitochondrial matrix is conducted by protein-mediated transport. Recently, the transporter SLC25A39 was shown to participate in GSH transport into the mitochondria and is upregulated by GSH depletion [13], suggesting that xenobiotic detoxification might impair its expression, affecting mitochondrial health. In response to different stimuli, including redox imbalance, the mitochondrial cell content and functionality are maintained by controlling mitochondrial biogenesis, degradation, and several other dynamic processes [14, 15]. Mitochondrial biogenesis is regulated by the Sirtuin 1 (SIRT1) — peroxisome proliferators-activated receptor-γ coactivator 1α (PGC1α) — nuclear respiration factor 1 (NRF1) signaling pathway, which control the transcription of nuclear-encoded mitochondrial proteins and the mitochondrial transcription factor A (TFAM) expression, the master regulator of mitochondrial-encoded protein expression and mitochondrial DNA replication [16, 17].

Liver damage induced by As or F^−^ exposure has been associated with oxidative damage induction through GSH depletion and inhibition of GSH-dependent enzymes. Recently, GSH depletion, inhibition of GSH peroxidase 4, and the increase in lipoperoxidation products, soluble ferrous iron levels, and p53 levels have been associated with ferroptosis, a type of cell death induced by cellular redox imbalance, and GSH loss [18]. In addition to hepatocytes, liver tissue comprises different cell types, including hepatic stellate cells (HSCs). The latter are resident fibroblasts, which are characterized by the expression of markers such as glial fibrillary acidic protein (GFAP) and are highly susceptible to oxidative stress. After activation, their morphology changes, and collagen is produced as part of the defense-regeneration response after a liver insult [19].

Previous studies have shown that chronic exposure to high levels of As and/or F^−^ has been associated with liver damage by oxidative stress and mitochondrial dysfunction. This condition is characterized by increased malonaldehyde and nitric oxide levels, diminished antioxidant enzymes and GSH levels, and impaired mitochondrial homeostasis [20–25]. However, investigations of the toxic effects of simultaneous exposure to both elements must be better understood because some results are contradictory. Moreover, the impact of combined exposure to both elements at human-relevant doses starting at gestation has not been explored.

Here, we investigated whether the hepatotoxic effects of As and F^−^ were more severe than the exposure to As or F^−^ using CD1 male mice chronically exposed to 2 mg As/L and 25 mg F^−^/L in drinking water since gestation. GSH levels, sulfur-containing amino acid transporter expression, transsulfuration pathway activity, and the expression levels of mitochondrial biogenesis-related proteins and ferroptosis biomarkers were evaluated 30 (P30) and 90 (P90) days after birth. Serum transaminase levels (ALT), GFAP expression, and liver tissue sections were examined for liver damage. Additionally, the impact of exposure on mitochondrial homeostasis was addressed by estimating the relative mtDNA/nDNA content.

## Materials and Methods

### Chemicals and Antibodies

We purchased all chemicals from Sigma‒Aldrich (St Louis, MO, USA) if not contrarily indicated, 99% pure methylarsonic acid (MMAV) disodium salt from Chem Service (West Chester, PA, USA); sodium borohydride from EM Science (Gibbstown, NJ, USA). Santa Cruz Biotechnology (Santa Cruz, CA, USA) provided the following primary antibodies for Western blots: SIRT1 (B-7, sc74465), PGCα1 (D-5, sc-518025), NRF1 (147.1, sc-101102), TFAM (F-6, sc-166965), MT-CO2 (D-5, sc-514489), GFAP (GA-5, sc-58766), and secondary antibody anti-mouse IgM (sc-2064). From Proteintech (Rosemont, IL, USA) Anti-SLC25A39 (14,963–1-AP) was purchased. Cell Signaling Technology (Danvers, MA, USA) provided Anti-SLC1A4 (8442 s) antibody and secondary goat anti-rabbit antibodies. Abcam (Cambridge, MA, EE. UU) provided anti-4-Hydroxynonenal antibody (ab46545) and anti-ATF-4 antibody (ab23760), and Invitrogen (Waltham, MA, USA), the secondary anti-mouse IgG antibody.

### Animals and Treatment

CD-1 mice (8–10 weeks old) were obtained from the Animal Care Facility at the Instituto de Investigaciones Biomédicas, UNAM. They were kept at 23–25 °C in 12 h light/dark cycles and 50–60% relative humidity. Mice had free access to water and food (Harlan 2018S Diet; Harlan, Indianapolis, IN, USA). The Animal Care Committee at Instituto de Investigaciones Biomédicas, UNAM authorized the experimental protocol. The exposure protocol started on gestational Day 0. Pregnant mice (*n* = 40) were separated at random into 4 groups: Control, As, F^−^, and As + F^−^. The untreated control group received iAs/F^−^-free drinking water, the As-exposed group received 2 mg/L As daily as sodium arsenite, the F^−^ group received 25 mg/L F^−^ daily as sodium fluoride, and the As + F^−^ group received both elements at the above-mentioned concentrations. The As and F^−^ concentrations used were selected following the levels of A and F^−^ frequently found in water wells and considering that rodents are 5–tenfold more resistant than humans. Treatment solutions were prepared daily in deionized water to prevent As oxidation. Mice weight was determined weekly. Offspring were separated by sex during postnatal Day 21. Male litters continued exposure protocols until P30 or P90, when mice were euthanized, and the upper right lobe of the liver was collected and immediately frozen and stored at − 70 °C or used freshly for some experiments. For histological studies, mice were anesthetized and intracardially perfused with a fixative solution (4% paraformaldehyde in phosphate buffer). All animal procedures followed the “Principles of Laboratory Animal Care” guidelines (NIH publication #85–23, revised 1985) and “Especificaciones técnicas para la producción, cuidado y uso de los animales de laboratorio (Clave NOM-062-ZOO-1999)” of the “Norma Oficial Mexicana de la Secretaría de Agricultura, Ganadería, Desarrollo Rural, Pesca y Alimentación (SAGARPA)” (published in August 2001).

### GSH Determination

GSH levels were measured using a microplate-adapted fluorometric o-phthalaldehyde (OPA) method [26]. Fresh tissue samples were homogenized in 10 volumes of buffer A (154 mM KCl, 5 mM DTPA, and 0.1 M K_2_PO_4_), then, an equal volume of buffer B (40 mM HCl, 10 mM DTPA, 20 mM ascorbic acid and 10% TCA) was added. The samples were centrifuged at 14,000 × g for 30 min, and supernatants were filtered using Millipore PTFE 0.45 μm filters. GSH levels were determined by fluorescence with 365 nm/430 nm (excitation/emission) filters in a DTX 800/880 Multimode Detector (Beckman Coulter, Fullerton, CA, USA).

### Western Blotting

Liver proteins were isolated by homogenizing 20 mg of tissue in RIPA buffer (50 mM Tris HCl, pH 7.55, 150 mM NaCl, 2 mM EDTA, 1 mM EGTA, 1 mM DTT, 2.5 mM NaPPi, 1% Triton X-100, 0.1% sodium deoxycholate, 1 mM glycerol 2-P, 1 mM Na_2_VO_4_, 1 mM PMSF and 10 mg/mL aprotinin/leupeptine), and centrifuged at 14,000 × g for 15 min at 4 °C. Supernatants were stored at − 80 °C until analysis. Protein concentrations were determined using the Bradford methodology (Bio-Rad, CA, USA) and an albumin standard curve (*R*^2^ > 0.98). For protein semiquantitative identification, 20 μg of protein was loaded in 10% or 15% SDS-acrylamide gels and transferred onto PVDF membranes (Bio-Rad Laboratories, Germany). Membrane blockage was performed using a Tris-buffered saline solution containing 5% Blotto and 0.1% Tween-20. Membranes were afterward incubated with the respective primary antibodies at 4 °C overnight. Total protein Ponceau staining was used as a protein loading control. Protein bands were revealed after incubation with HRP-linked secondary antibodies using the Amersham ECL Advance™ Western blotting Detection Kit (GE Life Sciences, RPN2232). A C-Digit scanner (LI-COR) was used to capture images, and then, they were analyzed using ImageJ software version 1.46r software (US National Institutes of Health, Bethesda, MD, USA). The analysis of protein levels was performed by normalizing the optical density of each band with respect to the loading control and represented in relation to the control group.

### Transsulfuration Pathway Activity

Cystathionine β synthase** (**CBS) and Cystathionase (CSE) activities were measured in liver homogenates based on the ability of these enzymes to generate H_2_S [26]. The assay was run using 300 µg of protein from the homogenized liver, and the reaction solution contained a final concentration of 10 mM L-Cys and 1 mM PLP in 1X PBS. DL-propargylglycine (CSE inhibitor) was used to estimate each enzyme’s contribution to the total activity. For this, protein samples were incubated for 30 min at 4 °C at a final concentration of 7.5 mM of inhibitor before incubation with the reaction solution. Dot spots were quantified using ImageJ software version 1.46r software (US National Institutes of Health, Bethesda, MD, USA).

### Cysteine Determination

For free cysteine determination, 20 mg of tissue was homogenized in 1 ml of 8% of perchloric acid solution. Cysteine levels were spectrophotometrically analyzed following the method described by Gaitonde [27].

### Mitochondrial DNA Content

Liver mtDNA content was measured by real-time PCR using Rotor-Gene Q (Qiagen) and SYBR® FAST Universal 2X qPCR Master Mix (KAPA Biosystems, MA, USA; cat. # KK4600). Whole-cell DNA was isolated using a DNeasy Blood and Tissue kit (Qiagen). Liver mtDNA content was related to the amount of nuclear DNA (nDNA) [28]. The PCR conditions were as follows: an initial heating/denaturalization at 94 °C for 3 min, followed by 40 cycles of 94 °C for 1 s, 63 °C for 10 s, and 72 °C for 12 s. The primers used were as follows: 5′-ctagaaaccccgaaaccaaa-3′ and 5′-ccagctatcaccaagctcgt-3′ for mMito genome; and 5′-atgggaagccgaacatactg-3′ and 5′-cagtctcagtgggggtgaat-3′ for Mβ2µglobulin (nuclear gene). The difference in the threshold cycle values between the nuclear and mitochondrial genes was used to estimate the relative abundance of the mitochondrial genome. The mtDNA/nDNA ratio is reported as 2^−ΔΔCt^ [29].

### Quantitative RT-PCR Analysis of ikkbα

Total RNA from the liver of mice was isolated using TRIzol (Invitrogen, Carlsbad, CA, USA). The integrity and purity of the RNA were assessed by the presence of 18S and 28S ribosomal RNA in 1% agarose gels and the absorbance indices A260/280 and A260/230. RNA concentrations were calculated by measuring the absorbance at 260 nm. Complementary DNA (cDNA) was synthesized from one microgram of total RNA using Moloney murine leukemia virus (M-MLV) reverse transcriptase and oligo(dT)15 primer (Promega, Madison, WI, USA). For quantitative PCR analysis, the cDNA of individual samples was diluted to 5 ng of input total RNA in a reaction mixture containing 0.25 μM of each respective forward and reverse primer and 1 × KAPA SYBR FAST Universal Mix (Kapa Biosystems, Cape Town, South Africa), the amplification was performed in a Rotor-Gene Q PCR cycler (Qiagen GmbH, Hilden, Germany). The PCR conditions were as follows: an initial heating/denaturalization at 94 °C for 3 min, followed by 40 cycles of 94 °C for 1 s, 63 °C for 10 s, and 72 °C for 12 s. Each PCR run generated melting curves from 70 to 95 °C. The primers used were as follows: 5′-aaatctccagatgctacccgagag-3′ and 5′-ataatgtcagacgctggcctccaa-3′ for *Iκκbα*; and 5′-caaatgctggagaagaatcggt-3′ and 5′-catcgacttctgcatgtttaggc-3′ for *Sdha,* as reference gene. The results were analyzed using the 2^−ΔΔCt^ method [29] and expressed as the mean normalized values ± SD.

### Free Fe^2+^ Content Determination

The levels of free Fe^2+^ were measured as a marker of ferroptosis in liver homogenates using an Iron Assay Kit (Colorimetric) (Abcam Cat No. ab83366). The absorbance of the complex formed by Fe^2+^ with the iron probe was read at 593 nm in a DTX 800/880 Multimode Detector (Beckman Coulter, Fullerton, CA, USA).

### GPX4 Activity Assay

The activity of GPX4 was assessed by a colorimetric NADPH-coupled assay using tert-butyl hydroperoxide as the substrate [30]. Briefly, 20 mg of liver tissue was homogenized in 250 µl of non-denaturing lysis solution (25 mM Tris Base, 1 mM DTT, 5% glycerol, 1% Triton X-100), followed by three freeze–thaw cycles. Finally, samples containing 20 μg of protein, 30 mM Tris HCl (pH 7.4), 3 mM EDTA, 0.2 mM NADPH, 1 mM GSH, and 0.2 U GSH reductase were placed in a 96-well plate at a final volume of 250 µl. The assay started with the addition of 20 µl of 1.5 mM tert-butyl hydroperoxide. The decrease of NADPH absorbance was monitored at 340 nm at 37 °C, using Synergy H4TM. GPX4 activity was calculated from the slope of the curves as µmoles NADPH per minute (U), considering the extinction coefficient of NADPH (E_NADPH_ = 6.22 mM^−1^ cm^−1^). The data were expressed in U/mg protein.

### Serum Transaminase Activity

Alanine aminotransaminase (ALT) activity was determined as a marker of liver damage by a microplate-adapted procedure using dinitrophenyl-hydrazine (DNPH) [31]. Blood samples were obtained from the submandibular venous sinus for further isolation of the serum fraction. ALT activity was determined using 10 µl of serum and expressed as U/L using the following equations: $EA \left(ALT\right)=\frac{\upmu mol(30min)-\upmu mol(0min)}{30min*0.00001L}$.

### Liver Tissue Section Histology

Liver tissue section analysis was performed at P90. Animals were perfused with a fixative solution (4% paraformaldehyde in phosphate buffer pH 7.4), and liver tissue was extracted for fixation in fresh 4% paraformaldehyde fixative solution for 24 h. Subsequently, the fixed tissue was washed 4 times in 1X PBS for 30 min, processed using Histo-Kinette, and embedded in paraffin. Tissue sections (4–5 µm) were collected on polylysine-coated slides and processed for hematoxylin–eosin (H&E) staining or Masson’s trichrome staining.

### Arsenic and Fluoride Level Determination

As species and F^−^ levels were determined in the upper right lobe liver tissue by hydride-generation atomic absorption spectrometry using cryotrapping (HG-CT-AAS) and isothermal distillation technique combined with a potentiometric method using an ion-selective electrode, respectively, as previously described [32]. Liver F^−^ was determined in pooled samples of 3 mice per group. The relative proportions of arsenic species (iAs%, MMA%, and DMA%) were calculated as $\frac{\left(\mathrm{each arsenic species }\times 100\right)}{\mathrm{total As content}}$. Arsenic methylation indices were calculated as MMAs/iAs (primary methylation index) and DMAs/MMAs (secondary methylation index) [33].

### Data Analysis

The data are expressed as the mean ± standard error. The number of individuals tested is indicated in each case. One-way analysis of variance (ANOVA) was used to assess statistical significance, followed by post hoc tests, as shown in the corresponding figures. A *p* < 0.05 was considered statistically significant in all cases.

## Results

### Oxidative Stress and Ferroptosis Biomarkers are Induced by the Combined Exposure to As and F^−^ at P30 but not at P90

**Fig. 1:**
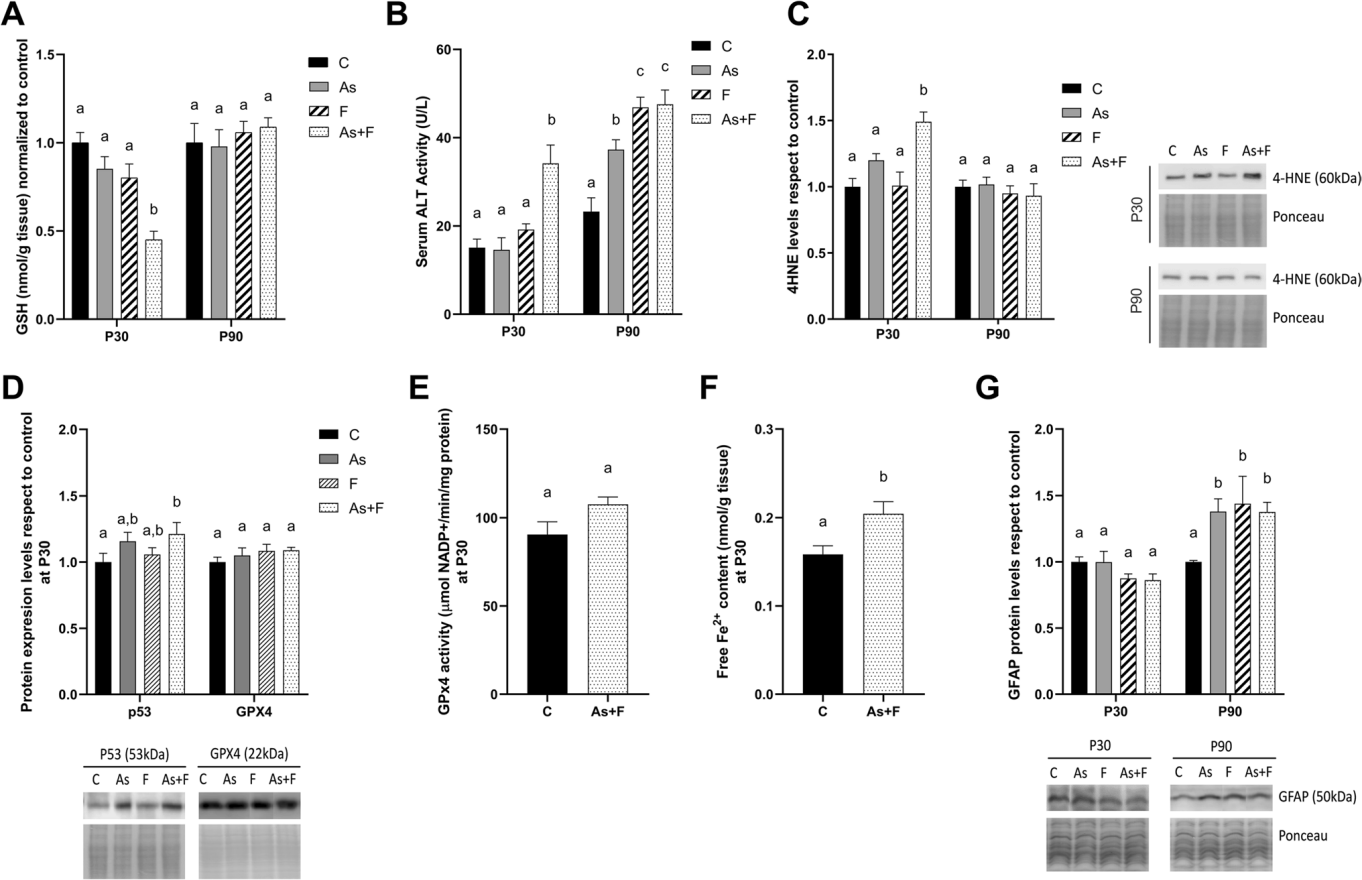
Oxidative damage, serum ALT, and ferroptosis biomarkers in male mouse liver. **A** GSH levels at P30 and P90. Bars show means ± SEMs with respect to controls. Data were analyzed by two-way ANOVA, followed by Tukey’s post hoc test (*N* = 7). **B** Serum alanine aminotransaminase (ALT) at P30 and P90. Bars show means ± SEMs. Data were analyzed by Kruskal–Wallis ANOVA followed by Dunn’s post hoc test (*N* = 10). **C** 4HNE-conjugated protein levels in the liver at P30 and P90. Bars show means ± SEMs of the densitometric determination of Western blot images normalized against total protein stain as a loading control and expressed with respect to controls. Data were analyzed by two-way ANOVA followed by Tukey’s post hoc test (*N* = 6). **D** Protein expression levels of p53 and GPX4 in the liver at P30. Bars show means ± SEMs of the densitometric determination of Western blot images normalized against total protein stain as a loading control and expressed with respect to controls. Data were analyzed by two-way ANOVA, followed by Tukey’s post hoc test (*N* = 6). **E** GPX4 enzymatic activity and (**F**) free ferrous iron in the liver at P30. Data were analyzed using Student’s* t* test (*N* = 6). Bars show means ± SEMs, normalized against controls. **G** Protein expression levels of GFAP. Bars show means ± SEMs of the densitometric determination of Western blot images normalized against total protein staining as a loading control and expressed with respect to controls. Data were analyzed by two-way ANOVA, post hoc analysis: Tukey’s test (*N* = 6). Different letters above each bar indicate significant differences among means. Significance level *P* < 0.05

The exposure protocol did not alter the weight gain and water consumption of pregnant mice or their litters (Fig. S1). The levels of GSH in the liver were slightly but not significantly reduced in the As- and F^−^-exposed groups compared to the control at P30. In contrast, the combined (As + F^−^)-exposed group showed a significant reduction in the content of the GSH (Fig. 1A). The marked decrease in GSH levels and increase in serum ALT at P30 (Fig. 1B) coincided with a significant increase in 4HNE conjugated to proteins, a marker of oxidative stress-induced damage (Fig. 1C). At the same time, significantly higher expression of p53 and free Fe^2+^ levels (Fig. 1D, F), which are considered ferroptotic biomarkers, were observed. However, the expression levels of GPX4 and its activity did not change significantly (Fig. 1D and E). Meanwhile, at P90, the serum levels of ALT and GFAP protein levels in the As-, F^−^- and As + F^−^-exposed groups were significantly higher than in the control groups (Fig. 1B, G), but the levels of 4HNE (Fig. 1C) and ferroptotic biomarkers (data not shown) were not altered in any group.

### As + F^−^ Exposure Induces Cysteine Uptake and Biosynthesis in Response to GSH Depletion

**Fig. 2:**
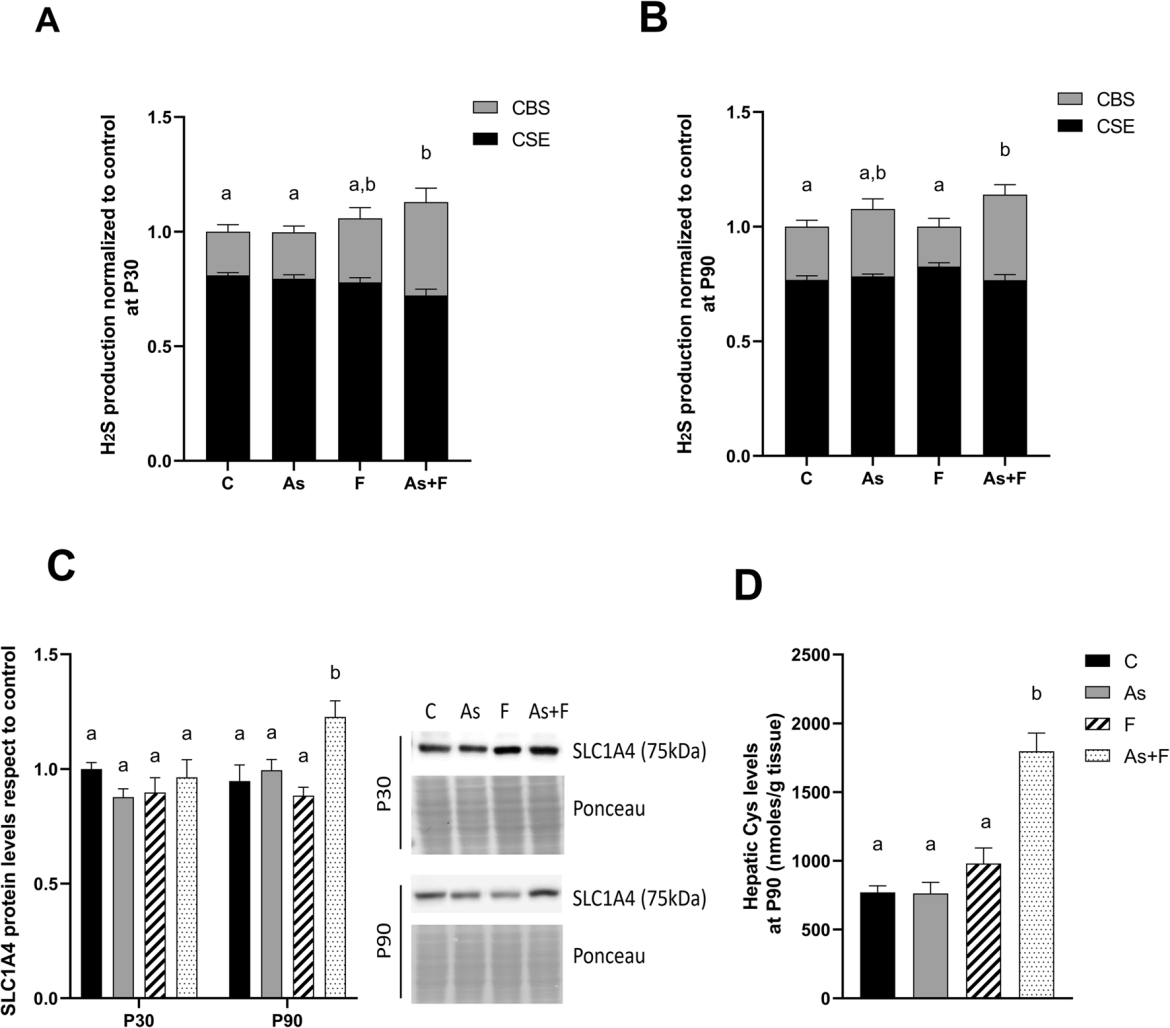
Cysteine synthesis and uptake are induced in the liver to recover GSH levels. Contribution of CBS and CSE activity to H_2_S production in the liver at P30 (**A**) and P90 (**B**). Bars show means ± SEMs of the densitometric determination of PbS spots formed during the enzymatic reaction. D-propargylglycine inhibitor was used to estimate the specific activity of each enzyme. Data were analyzed by two-way ANOVA followed by Tukey’s post hoc test (*N* = 6). **C** Protein expression levels of cysteine transporter SLC1A4. Bars show means ± SEMs of the densitometric determination of Western blot images normalized against total protein staining as a loading control and expressed with respect to controls. Data were analyzed by two-way ANOVA, post hoc analysis: Tukey’s test (*N* = 6). **D** Free cysteine levels in the liver at P90. Bars show means ± SEMs with respect to controls. Data were analyzed by one-way ANOVA followed by Tukey’s post hoc test (*N* = 6). Different letters above each bar indicate significant differences among means. Significance level *P* < 0.05

The recovery of GSH levels in the As + F^−^-exposed group at P90 was associated with a sustained increase in the activity of CBS in the transsulfuration pathway at both P30 and P90 (Fig. 2A and B). The levels of SLC1A4 transporter protein and cysteine content in the As + F^−^-exposed group at P90 were significantly increased (Fig. 2C and D).

### Mitochondrial Protein Expression and Mitochondrial DNA Content are Altered by As + F^−^ Exposure

**Fig. 3:**
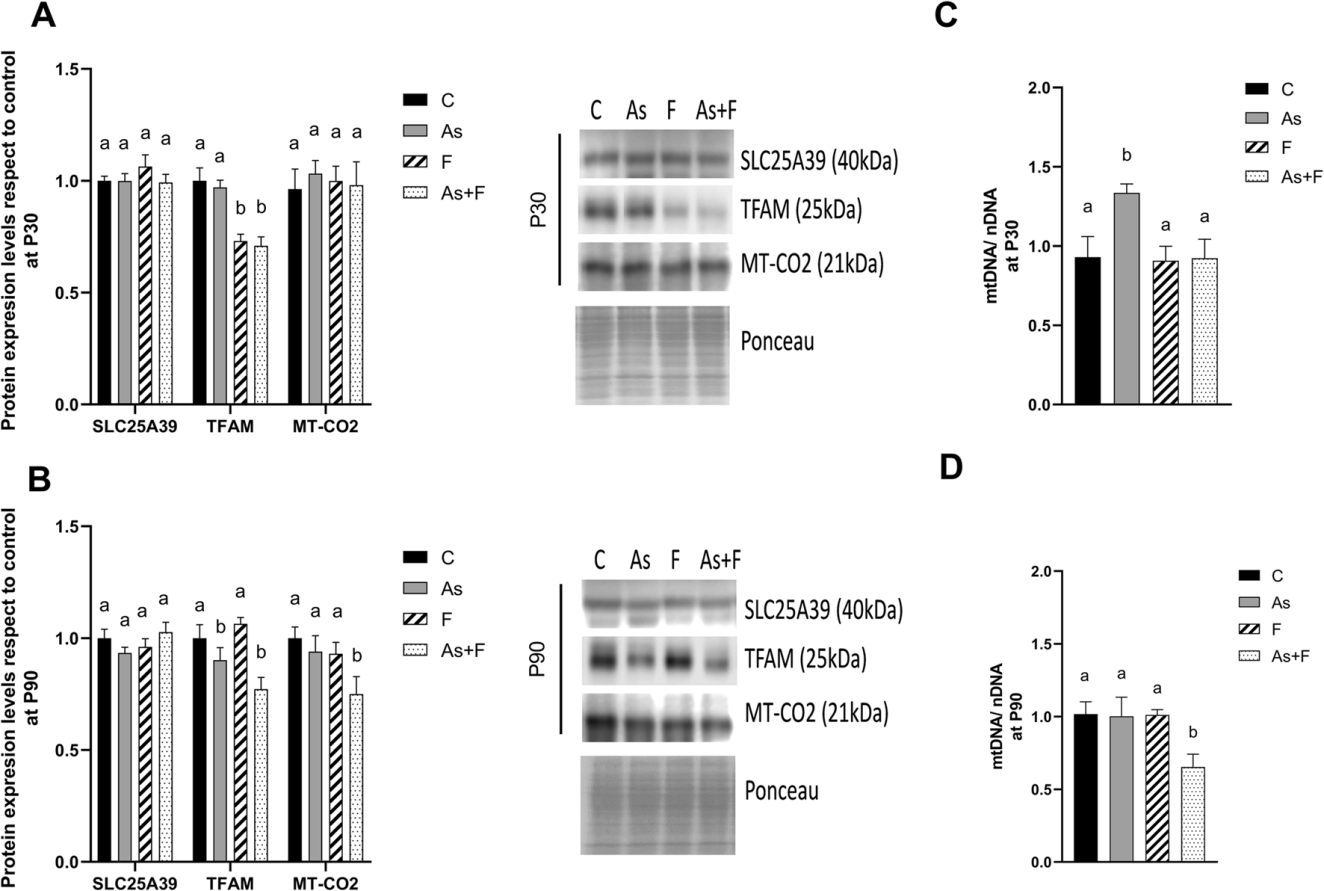
Mitochondrial protein expression levels and mt-DNA/nDNA are altered in the livers of mouse offspring after As + F^−^ exposure at P90. Protein expression of mitochondrial proteins: SLC25A39, TFAM and MT-CO2 at P30 (**A**) and P90 (**B**). Bars show means ± SEMs of the densitometric determination of Western blot images normalized against total protein stain as loading control and expressed with respect to controls. Data were analyzed by two-way ANOVA, followed by Tukey’s post hoc test (*N* = 6). The relative content of mitochondrial DNA was measured as mt-DNA/nDNA at P30 (**C**) and P90 (**D**). Bars show means ± SDs of 2^−ΔΔCt^ values. Data were analyzed by two-way ANOVA, followed by Tukey’s post hoc test (*N* = 6). Different letters above each bar indicate significant differences among means. Significance level *P* < 0.05

**Fig. 4:**
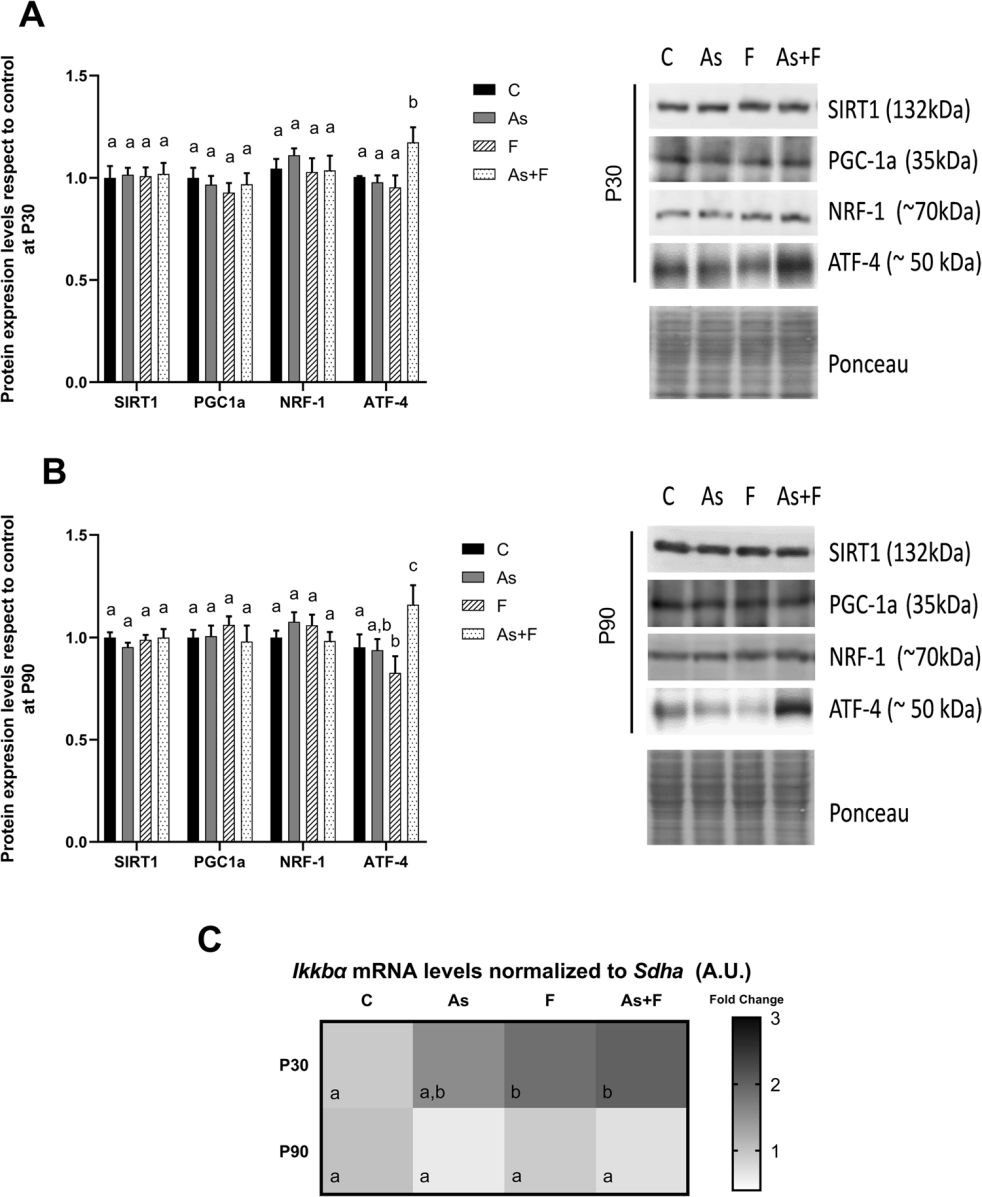
The Signaling pathway controlling mitochondrial biogenesis and TFAM transcription are differentially regulated in the livers of mouse offspring after As + F exposure at P30 and P90. Expression of mitochondrial biogenesis-related regulator proteins at P30 (**A**) and P90 (**B**). Bars show means ± SEMs of the densitometric determination of Western blot images normalized against total protein staining as a loading control and expressed with respect to controls. Data were analyzed by two-way ANOVA, followed by Tukey’s post hoc test (*N* = 6). **C** Heatmap representation of *Ikkbα* mRNA level in the liver at P30 and P90 measured by qRT-PCR. Data were analyzed by two-way ANOVA, followed by Tukey’s post hoc test (*N* = 6). The color gradient was used as representative of fold changes in the expression. *Sdha* transcript was used as a reference gene. Different letters above each bar indicate significant differences among means. Significance level *P* < 0.05

We explored the expression levels of the proteins involved in the mitochondrial homeostasis and biogenesis signaling pathway. The protein expression levels of SLC25A39 were not modulated by the exposure to As or/and F^−^ at either of the times evaluated (Fig. 3A, B). However, the expression of TFAM was significantly reduced at P30 in the F^−^- and As + F^−^-exposed groups, and at P90 in the As- and As + F^−^-exposed groups (Fig. 3A, B). TFAM is a transcription factor that binds mtDNA for gene transcription and mitochondrial genome replication [34]. Interestingly, the relative content of mtDNA/nDNA was not affected by exposure at P30, except for the animals exposed to As (Fig. 3C). In contrast, the protein expression levels of MT-CO2, a mitochondrial-encoded subunit of the cytochrome C oxidase complex, were significantly reduced in the As + F^−^-exposed group (Fig. 3B), as was the relative content of mtDNA/nDNA (Fig. 3D) at P90. TFAM transcription is positively regulated by PGC1α/NRF1 and negatively by nuclear factor kappa-light-chain-enhancer of activated B cells (NF-kB) and activating transcription factor 4 (ATF-4) [35, 36]. We did not observe changes in the expression levels of the proteins upstream of TFAM from canonical pathway: SIRT1, PGC1α, and NRF1 at P30 or P90 (Fig. 4A, B), but the levels of *Ikkbα* mRNA were increased in F^−^ and As + F^−^ groups at P30 (Fig. 4C). ATF-4 upregulation was observed in As + F^−^ group at both moments, whereas ATF-4 was downregulated in F^−^ group at P90 (Fig. 4A, B).

### As + F^−^ Exposure Induces Histological Alterations and Fibrotic Initiation

**Fig. 5:**
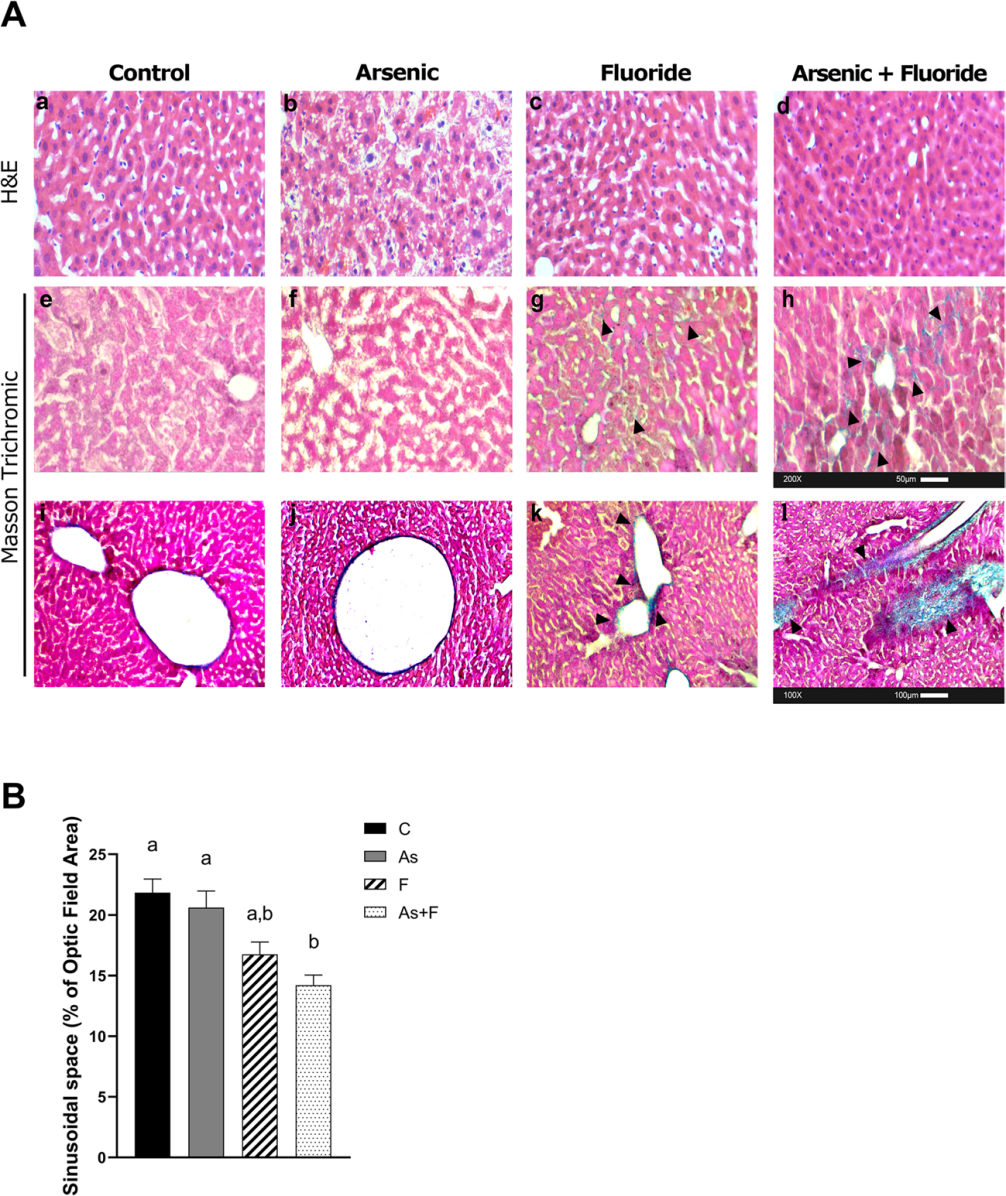
Hepatic histopathological alteration is induced by As + F exposure at P90. *A* Representative images of histological Studies H&E at 200 × (**a**–**d**) and Masson trichrome staining at 200 × (**e**–**h**) and 100 × (**i**–**l**). Black-head arrows show disarranged collagen fibers (stained in blue). *B* Percent of the sinusoidal area at P90. H&E histology (200 ×). Bars show the means ± SEMs. Data were analyzed by Kruskal–Wallis ANOVA, followed by a Dunn’s post hoc test (*N* = 4 mice/group, 40 optic fields per mouse). C: controls, As: Arsenic-exposed, F: Fluoride-exposed, and As+F: Arsenic+Fluoride-exposed groups. Different letters above each bar indicate significant differences among means. Significance level *P* < 0.05

H&E-stained liver tissue sections showed the presence of increased vacuolation inside the cytoplasm in the As-exposed mice as compared with controls (Fig. 5A-b), while a substantial reduction in the sinusoidal areas was observed in the As + F^−^-exposed group (Fig. 5A-d, B). Moreover, Masson trichrome-stained liver sections showed collagen fibers only delimited to large vessels, with large fibers arranged longitudinally surrounding the vessels in control mice, whereas in F^−^- and As + F^−^-exposed groups, the collagen fibers were disarranged, with increased prolongation into the cellular compartment, surrounding even smaller vessels, and not limited to vessel space (Fig. 5A-g, h, k, l). Collagen fiber aggregation was more frequent in the As + F^−^-exposed group than in the As- and F^−^-exposed groups. Since the upregulation of GFAP was observed in all exposed groups at P90, the activation of HSCs in As + F^−^-exposed mice possibly started earlier than that in As- and F^−^-exposed groups.

### As + F^−^ Exposure Alters the As Methylation Profile in the Liver

The levels of iAs among groups were similar at P30 or P90. Methylated As species were not detected in the control and F^−^-exposed groups at P30, and low levels were detected at P90. The levels of methylated As species (MMA + DMA) in the As- and the As + F^−^-exposed groups, were not significantly different at P30 or P90 (Table 1). However, a marked significant reduction in the percentage of DMA species and the DMA/MMA coefficient (Table 1) was observed at P90 in the As + F^−^-exposed group compared to the As-exposed group, similar to what has been previously reported [33, 37]. Concerning the F^−^ levels at P90, we observed increased content in the F^−^- and As + F^−^-exposed groups, compared to control and As-exposed groups. (C: 0.0126 ± 0.002 µg/g, As: < 0.01, F: 0.059 ± 0.056 µg/g, As + F^−^: 0.062 ± 0.023 µg/g).

**Table 1:** Concentrations of arsenic species in the liver of male mice offspring at P30 and P90

	Control	Arsenic	Fluoride	Arsenic + Fluoride
		(As)	(F^−^)	(As + F^−^)
	**P30**			
iAs (ng/g)	33.62 ± 29.32	30.47 ± 14.42	37.24 ± 11.94	27.85 ± 9.56
MMA + DMA (ng/g)	< LOD	16.79 ± 1.59	< LOD	59.07 ± 49.67
%iAs	98.4 ± 1.31	62.16 ± 10.27	99.38 ± 0.4	44.39 ± 14.32
%MMA	< LOD	10.99 ± 6.89	< LOD	22.03 ± 11.64
%DMA	< LOD	19.33 ± 12.53	< LOD	33.59 ± 20.82
MMA/iAs	< LOD	0.23 ± 0.09	< LOD	0.89 ± 0.7
DMA/MMA	< LOD	1.778 ± 0.45	< LOD	1.41 ± 0.44
	**P90**			
iAs (ng/g)	8.78 ± 5.47	9.26 ± 2.93	12.93 ± 3.79	15.9 ± 13.13
MMA + DMA (ng/g)	0.99 ± 0.45	5.08 ± 0.27	1.07 ± 0.82	4.632 ± 1.78
%iAs	75 ± 13.44	63.25 ± 8.08	89.08 ± 7.70	73.32 ± 13.53
%MMA	9.36 ± 5.32	9.92 ± 2.24	9.79 ± 4.79	10.65 ± 6.49
%DMA	13.33 ± 7.68	26.83 ± 6.31	3.86 ± 3.28	16.03 ± 8.84 *
MMA/iAs	0.14 ± 0.19	0.16 ± 0.06	0.11 ± 0.06	0.16 ± 0.11
DMA/MMA	0.21 ± 0.79	2.74 ± 0.47	0.20 ± 0.09	1.77 ± 0.81 *

*iAs*, inorganic arsenic; *MMA*, monomethylarsonic species; *DMA*, dimethylarsonic species level. Mice received purified water (control), As (2 ppm), F^−^ (25 ppm) and As + F^−^ (2:25 ppm) by drinking water for GD0-P30 or GD0-P90 days. For statistical purpose, limit of detection (LOD) were (ng/g) iAs = 0.126, MMA = 0.130 y DMAs = 0.144. The results are expressed as mean ± SD (*n* = 5). Data were analyzed using the Student’s *t*-test between As and As + F^−^ groups. * *p* < 0.05

## Discussion

As and F^−^ are the most frequent natural chemical pollutants in drinking water worldwide and continue to pose a health burden in countries that struggle to follow the health-based values recommended by the WHO (2021). Chronic exposure to these elements impacts children’s and adult’s health. Depending on the exposure dose and time, it has been associated with osteo- and neuropathies and skin, lung, and liver damage in populations worldwide [34].

Liver fibrosis is an abnormal accumulation of extracellular matrix proteins in response to cell damage and is considered an initial event in chronic liver diseases [38]. Here, we observed that male CD1 mice chronically exposed to As + F^−^ by drinking water since gestation showed liver cell damage biomarkers and liver fibrosis. Early after lactation (P30), a significant reduction in GSH levels was detected in the As + F^−^-exposed group, which coincided with an increase in serum ALT, and biomarkers of ferroptosis, such as increased 4HNE, free Fe^2+^, and p53 protein (Fig. 1C, D and F). Similarly, in other studies, F^−^ can also activate p53-dependent ferroptosis cell death in the liver, in response to redox and mitochondrial imbalance [39].

Later (P90), liver GSH levels in As + F^−^-exposed animals were similar to control levels. This condition was associated with increased cysteine levels (Fig. 2D) that could result from increased uptake by the upregulation of SLC1A4 (Fig. 2C) and its biosynthesis (Fig. 2A and B). The increased levels of GSH observed at P90 compared to those at P30 probably reduced lipid peroxidation (Fig. 1C) and ferroptosis biomarkers, suggesting that initial oxidative stress at P30 induced an antioxidant response that led to GSH synthesis. It has been shown that cysteine transporter and transsulfuration enzyme expression is modulated by nuclear factor erythroid 2-related factor 2 (NRF2) or ATF-4 in response to oxidative stress and amino acid limitation [40, 41]. Moreover, ROS also regulates the activity of enzymes involved in the entrance of homocysteine into the transsulfuration pathway by inhibiting its remethylation [42]. The upregulation of the SLC1A4/ASCT1 cysteine transporter in the liver after acetaminophen-induced GSH depletion [43] and increased Hcy flux through the transsulfuration pathway [44] has been previously reported.

Notwithstanding the antioxidant response at P90, elevated serum ALT activity, upregulation of GFAP, and a significant deposition of collagen fibers were observed, suggesting that HSC activation leads to a fibrotic reaction and tissue damage. An increase in GFAP expression is considered an early marker of HSC activation induced by liver insult [45]. HSC can be activated by ROS, lipid peroxides, and damage-associated molecular patterns molecules released by hepatocytes during liver injury [19, 45]. Oxidative stress is the primary mechanism of cytotoxicity induced by different chemicals, As or F^−^. Moreover, redox imbalance caused by As or F^−^ promotes mitochondrial/endoplasmic reticulum stress and inflammation [46, 47], which could also prime HSC [48]. An increase in HSC activation markers often occurs along with a reduction in GSH and other antioxidant components after prolonged exposure to carbon tetrachloride (CCl4), thioacetamide (TAA), or As [49–52]. In this model, even though GSH levels did not change, and no ferroptosis signal was documented at P90, the mitochondrial dysfunction observed could contribute to the increased GFAP in the As + F-group. The alterations in mitochondrial protein expression in As + F^−^ group, were observed since P30, coinciding with biomarkers of oxidative stress. Conversely, the increase in GFAP and ALT activity at P90 in the case of As or F^−^ alone exposure without increased collagen fiber deposition suggests that the process could have a delayed onset compared to As + F^−^. However, our current data are insufficient to support that mitochondrial dysfunction and oxidative stress are involved in HSC activation in As or F^−^ individual exposure in this model.

As mentioned before, another piece of evidence for tissue damage is the maintained downregulation of TFAM in the As + F^−^ group (P30 and P90, Fig. 3A and B). TFAM is regulated by redox and energy status through canonical and non-canonical pathways by PGCα1/NRF1, SP1, NFkB, and ATF-4 [35, 36]. NF-kB and ATF-4 repress TFAM transcription, and the activation of NF-kB or ATF-4 has been associated with As or F^−^ -induced oxidative stress. Here, we did not observe changes in the expression of proteins from the canonical pathway, but changes in their posttranslational modification and activity shouldn’t be ruled out as possible mechanisms involved in the modulation of TFAM. Dong et al. (2020) showed an increased ATF-4 expression in As- and As + F^−^-exposed groups at P90 in the liver but not in the F^−^ group in a gestational exposure model in rats [53]. Here, we saw a significative increment in *Ikkb*α mRNA, a product of NF-kB activation, at P30 in the F^−^ and As + F^−^ groups. At the same time, ATF-4 protein was also upregulated in the As + F^−^ group at P30 and P90, suggesting the participation of both factors in the repression of TFAM in the combined group. Moreover, ATF-4 levels decreased in the F^−^ group at P90, which coincides with the recovery of TFAM levels at this time. Thus, time-dependent effects on the activation of these factors by As or F^−^ could explain the temporal difference in the modulation of TFAM observed in the individual exposures.

TFAM reduction affected the expression of MT-CO2 and mDNA/nDNA content at P90 (Fig. 3B and D). The downregulation of TFAM has been associated with a significant reduction in the mtDNA/nDNA content [16]. Most mitochondrial genes are encoded in nuclear DNA and are under the transcriptional control of PGC1a and NRF1 [17]. However, mouse mtDNA encodes two ribosomal RNAs, 22 transference RNAs, and 13 mitochondrial proteins [54]. These proteins are involved in the mitochondrial electron transfer chain and are polycistronically transcribed under TFAM control [55]. Among them, MT-CO1 and MT-CO2 are part of the catalytic core of the cytochrome C oxidase complex (CcO, complex IV). MT-CO2 protein has the cytochrome C binding site and the two-copper CuA center, responsible for initiating electron transference from cytochrome C to O_2_. It is part of the H^+^ transport pore contributing to mitochondrial membrane potential [56]. Although CcO is not a source of ROS generation in mitochondria, as the last and rate-limiting complex involved in the electron transfer chain (ETC), it could alter the proper function of the earlier complexes. Altered expression and defects of MT-CO2 alter the balance among subunits in CcO and could harm ETC, ATP production and loss of mitochondrial membrane potential [56, 57]. Therefore, altered expression of TFAM alters the expression of MT-CO2 and the relative mtDNA/nDNA content and could impair the expression of other mitochondrial-encoded proteins of the ETC. We consider that evaluation of the effect of As + F^−^ exposure on other processes involved in mitochondrial homeostasis as well as mitochondrial activity studies, should be performed to understand better the effects of As + F^−^ exposure on mitochondrial function. Chemical-induced hepatotoxicity is often characterized by mitochondrial dysfunction associated with impaired biogenesis and dynamic activity of the ETC complex and loss of mitochondrial potential. Moreover, some of these chemicals induce fibrotic processes, as is the case for rifampin, isoniazid, TAA, and CCL_4_ [58–61], demonstrating the role of mitochondrial dysfunction in the pathology of liver fibrosis.

In addition, redox imbalance and altered sulfur-containing amino acid metabolism have been associated with fibrotic processes [62]. Activating the transsulfuration pathway or administrating H_2_S rescues mitochondrial function and alleviates liver pathogenic conditions and fibrogenesis [63, 64]. In contrast to previous evidence, we observed a positive modulation in the transsulfuration pathway in the mice exposed to the combination but impaired mitochondrial homeostasis and fibrotic process. The transsulfuration pathway is essential for synthesizing cysteine and H_2_S. However, in different conditions, the synthesis of one of both products may be favored based on the differential modulation of each enzyme in the pathway. It has been proposed that CSE activity rather than CBS is associated with the production of H_2_S in the liver, and this product has a protective role in mitochondrial homeostasis and against fibrosis [65, 66]. In our work, we observed an increase in the activity of CBS and the cysteine levels, which suggests that this product could be favored over H_2_S. Therefore, this differential modulation of transsulfuration pathways could explain why even increasing the transsulfuration pathway activity in the As + F- group did not protect against mitochondrial imbalance and led to fibrosis.

In contrast to F^−^ exposure, liver fibrosis was observed in humans and animals exposed to As [4, 49, 62]. Moreover, fibrosis is often an early event for carcinogenesis, and As gestational exposure induces hepatocellular carcinoma at a longer exposure time [67]. However, in rodent models, liver fibrosis has been observed at longer exposures where age could also play a role. In our study, F^−^-coexposure may aggravate As-induced liver injury and shorten the exposure time for the pathological manifestations compared to single-element exposure.

Another interesting observation during As methylation was the significant reduction in the percentage of DMA and, thus, a decline in the second methylation index (DMA/MMA) in the As + F^−^ coexposure group compared to the As group at P90, suggesting a biochemical interaction in the disposition of these elements that leads to a diminished As methylation by F^−^ coexposure. Studies of combined exposure to As + F^−^ have also observed impairment in As metabolism by F^−^ and a lower tissue accumulation of both elements. These studies used higher doses or shorter exposure times than our model [33, 37, 68]. A lower methylation rate could be due to transsulfuration pathway activation, which is linked to the transmethylation cycle and SAM pools. The increased flux into this metabolic pathway toward cysteine synthesis could compromise SAM’s availability for its use during iAs methylation. Moreover, F^−^ could impair As methylation by changes in AS3MT expression [69].

Finally, this study provides evidence that the adverse effects in the liver of the combination of As and F^−^ are more potent, especially in mitochondria, compared to As or F^−^ exposure alone. It was also observed that individual and combined exposure activated different adaptative response mechanisms. However, we considered that more studies are needed to investigate the signals involved in liver mitochondrial dysfunction and the fibrotic response.

## Conclusion

Male mice chronically exposed to As + F at low doses since gestation showed liver damage described initially by biomarkers of oxidative stress, such as reduced levels of GSH, increased lipid peroxidation, and biomarkers of ferroptotic processes that progressed into mitochondrial damage, HSC activation, and fibrotic deposition of collagen fibers suggesting the early initiation of fibrotic liver disease, but not in independent As or F exposure.

## Supplementary Information

Below is the link to the electronic supplementary material.

Supplementary file1 (TIF 3907 KB) Fig. 1S Effect of exposure on water consumption and body weight of offspring during the exposure period. A) Mean of daily water intake along the exposure period: gestation, lactation, and litter´s growth (pregnant mice *N*=10, offspring until P30 *N*= 40, offspring until P90 *N*=25) B) Body weight of male offspring along the exposure period, from P7 to P90 (*N*=25). Dots show means ± SEMs. Data were analyzed by two-way ANOVA, followed by Tukey's post hoc test.

## Data Availability

No datasets were generated or analysed during the current study.
